# Butyrate limits the replication of porcine epidemic diarrhea virus in intestine epithelial cells by enhancing GPR43-mediated IFN-III production

**DOI:** 10.3389/fmicb.2023.1091807

**Published:** 2023-01-20

**Authors:** Haiyan He, Xuelei Fan, Haiyan Shen, Hongchao Gou, Chunhong Zhang, Zhicheng Liu, Bin Zhang, Nile Wuri, Jianfeng Zhang, Ming Liao, Letu Geri

**Affiliations:** ^1^College of Veterinary Medicine, Inner Mongolia Agricultural University, Hohhot, China; ^2^Institute of Animal Health, Guangdong Academy of Agricultural Sciences, Guangzhou, China; ^3^College of Veterinary Medicine, South China Agricultural University, Guangzhou, China; ^4^Maoming Branch Center of Guangdong Laboratory for Lingnan Modern Agricultural Science and Technology, Maoming, China; ^5^Key Laboratory of Livestock Disease Prevention of Guangdong Province, Scientific Observation and Experiment Station of Veterinary Drugs and Diagnostic Techniques of Guangdong Province, Ministry of Agriculture and Rural Affairs, Guangzhou, China

**Keywords:** porcine epidemic diarrhea virus, butyrate, GPR43, intestinal epithelial cells, IFN-III

## Abstract

Porcine epidemic diarrhea virus (PEDV) is a threat to the health of newborn piglets and has a significant impact on the swine industry. Short-chain fatty acids (SCFAs) are gut microbial metabolites that regulate intestinal function through different mechanisms to enhance the intestinal barrier and immune function. In this study, we aimed to determine whether butyrate displayed a better effect than other SCFAs on limiting PEDV replication in porcine intestinal epithelial cells. Mechanistically, butyrate treatment activated the interferon (IFN) response and interferon-stimulated gene (ISG) expression. Further experiments showed that inhibition of GPR43 (free fatty acid receptor 2) in intestinal epithelial cells increased virus infection and reduced antiviral effects through IFN λ response. Our findings revealed that butyrate exerts its antiviral effects by inducing GPR43-mediated IFN production in intestinal epithelial cells.

## 1. Introduction

Porcine epidemic diarrhea virus (PEDV) is a member of the family Coronaviridae, and is a single-stranded, enveloped, positive-sense RNA virus. PEDV can infect pigs at variously ages with no seasonal differences. PEDV infection has been responsible for watery diarrhea with 80–100% mortality in neonatal suckling piglets (Sun et al., 2012; Thomas et al., 2015). Since 2010, PED has led to severe economic losses to the swine industry worldwide (Turlewicz-Podbielska and Pomorska-Mól, 2021), especially in Asian countries, such as China and South Korea, and in North America (Sekhon et al., 2016). PEDV is mainly transmitted *via* the fecal-oral route, although eating contaminated feed causes transmission to piglets, and nasal-oral transmission has also been reported (Dee et al., 2014; Li et al., 2018). Vaccines are an efficient approach to protect pigs from infection. However, vaccine protection against emerging highly virulent strains is unsatisfactory due to the high rate of PEDV genome mutation (Lin et al., 2019). PEDV has developed various strategies to escape innate immunity, including suppression of type I interferon and type III interferon production *via* PEDV structural and nonstructural proteins (Ding et al., 2014; Wang et al., 2016; Zhang et al., 2018). Therefore, developing economical and efficient treatments to reduce PEDV infection is desirable.

The gut microbiota plays a key role in host physiology and pathology. Recent evidence has highlighted the impact of the gut microbiota on enterovirus infection, including effects on tissues and organs beyond the gastrointestinal tract during viral infection, providing protection that activates the innate immune system required for antiviral immunity (Abt et al., 2012; Pfeiffer and Virgin, 2016). Short-chain fatty acids (SCFAs) are a class of synthesized bacterial metabolites derived from indigestible carbohydrates in the gut, which include acetate, propionate, and butyrate among the essential metabolites. Different dietary patterns can affect the gut microbiota composition and the concentration of SCFAs, and feeding mice a high fiber diet to protect against Influenza caused lung tissue failure and vascular leakage (Trompette et al., 2018). While, another study revealed that high-fiber feed protects mice against Respiratory syncytial virus (RSV) infection. Analysis of the fecal microbiota composition discovered that phylum Firmicutes, related to the production of SCFAs, was markedly increased (Antunes et al., 2019). Butyrate promotes the antiviral effect of IFN when the encephalomyocarditis (EMC) virus infects MSV cells, while, butyrate protects against more severe disease caused by viral infection *in vivo* (Pouillart et al., 1992). SARS-CoV-2 is an enveloped, single-stranded positive-sense RNA which is member of coronavirus family. Clinical characteristics of COVID-19 shown as fever, dry cough, dyspnea, septic shock, coagulation dysfunction and multiple organ dysfunction or failure (Wang et al., 2020). Jardou and Lawson (2021) was pointed that butyrate supplementation for preventing the cytokine storm facilitate excessive activation of immune system and further development of disease. Butyrate contributes to resistance against viral in the lung that increased resistance against respiratory viral infection with lower respiratory tract infection (LRTI) (Haak et al., 2018). Butyrate affects viral infection by affecting the expression of certain genes. For instance, butyrate upregulates expression of the coxsackievirus and adenovirus receptor (CAR), which is the receptor for coxsackievirus B3 (Küster et al., 2010).

Accumulating evidence indicates that SCFAs are associated with a variety of diseases, including Inflammatory bowel diseases (IBD) (Parada Venegas et al., 2019), chronic kidney disease (Magliocca et al., 2022), colorectal cancer (CRC) (Hou et al., 2022), obesity (Alsharairi, 2021), and diabetes (Puddu et al., 2014). SCFAs can regulate fluid absorption in the colon and improve the efficacy of oral rehydration solution (ORS) to treat acute diarrhea (Ramakrishna et al., 2000). In addition, to enhance colonic fluid production, SCFAs can strengthen the mucosal barrier and improve the immune response after bacterial infection (Ranjbar et al., 2021). Poelaert et al. (2019) described that SCFAs could reduce Equine herpesvirus 1 transfer and infection *via* downregulation of endothelial cell adhesion molecules. SCFAs regulate the host immune system and stress by activating G protein-coupled receptors (GPCRs), which affect host metabolic pathways, and inhibit histone deacetylases (Martin-Gallausiaux et al., 2021). GPR41 (free fatty acid receptor 3 (FFAR3)), GPR43 (FFAR2), and GPR109a (G-protein coupled receptor 109A) are the major host receptors for SCFAs, all of which are expressed in various cell types, including intestinal cells and immune cells. A series of studies demonstrated that GPR43 plays an important role in the ‘gut-lung axis’, which can improve the respiratory tract’s protection by ligand acetate treatment when infected by microorganisms (Galvão et al., 2018; Antunes et al., 2019). GPR109a, also known as hydroxycarboxylic acid receptor 2 (HCA2), is not a receptor for nicotinate (niacin) but can be activated by high concentrations (half maximal effective concentration (EC_50_) approximately 1.6 mM) of sodium butyrate (Thangaraju et al., 2009).

PEDV infection reduced the abundance of some beneficial bacteria such as *Ruminococcaceae* and *Butyricimonas* (SCFA-producing bacteria species in the gut) and increased the abundance of *Firmicutes* and *Proteobacteria* (Huang et al., 2019). Several experiments demonstrated that adding medium-chain fatty acids (MCFAs) into feed effectively reduces PEDV infection (Jackman et al., 2020). However, the effect of gut microbiota metabolites on PEDV-infected intestine epithelial cells and antiviral responses has not been evaluated. In the present study, we aimed to determine the antiviral role of sodium butyrate on PEDV. The results showed that sodium butyrate, *via* the activation of GPR43, modulated the interferon response in intestine epithelial cells, thus protecting against PEDV infection.

## 2. Materials and methods

### 2.1. Cell lines and virus

African green monkey (Vero) cells were cultured in Dulbecco’s modified Eagle’s medium (DMEM) (Gibco™, Grand Island, NY, United Sttaes; 119,955) supplemented with 5% fetal bovine serum (FBS) (Gibco™; 10,099). Porcine intestinal epithelial (IPEC-J2) cells were cultured in Roswell Park Memorial Institute (RPMI) Medium 1,640 (Gibco™; 118,755) supplemented with 10% FBS. PEDV strain GD/HZ/2016 (GenBank Accession: OP191700.1) was preserved in our laboratory.

### 2.2. Biochemical reagents and antibodies

The GPR43 inhibitor GLPG0974 (SML2443), acetate (S2889), propionate (P5436), and butyrate (V900464) were purchased from Sigma-Aldrich (St. Louis, MO, United States). The GPR109a inhibitor Mepenzolate bromide (MPN) (HY-17585) and the nuclear factor kappa B (NF-κB) pathway inhibitor BAY 11–7,082 (HY-13453) were obtained from MedChemExpress (Monmouth Junction, NJ, USA). The primary antibodies for western blotting were mouse monoclonal antibodies against PEDV N (Medgene Labs, Brookings, SD, USA; 1,403,113) and glyceraldehyde-3-phospahte dehydrogenase (GAPDH) (ABclonal, Wuhan, China; AC002). The secondary antibodies used for western blotting were horseradish peroxidase (HRP)-Goat Anti Mouse IgG (H + L) (EARTH OX, Millbrae, CA, United States; E030110). The Alexa Fluor 488-conjugated goat anti-rabbit IgG (H + L) was purchase from Thermo Fisher Scientific (Waltham, MA, United States; A-11001).

### 2.3. Cytotoxicity assays

Cell viability was assessed using Cell Counting Kit-8 (Beyotime, Jiangsu, China; C0038) following the manufacturer’s instructions. IPEC-J2 cells in medium were seeded in 96-well plates at a density of 5,000 cells/well. Overnight incubation, the cells were treated with butyrate concentrations for 48 h. Thereafter, 10 μl of the CCK-8 reagent was added to each well, and the cells were further incubated at 37°C for 2 h. The absorbance was measured at 450 nm using a microplate reader (Thermo Fisher Scientific).

### 2.4. RNA interference

A *GPR43*-specific shRNA (GGCTGTGGTGACACTCCTTAACTCGAGTTAA-GGAGTGTCACCACAGCC) and negative control shRNA (shNC) designed by Cyagen (Santa Clara, CA, United States) were used to knock down *GPR43*. IPEC-J2 cells were transfected using Lipo8000™ Transfection Reagent (Beyotime; C0533) as outlined in the manufacturer’s protocol. In brief, IPEC-J2 cells were seeded in 12-well plates at a density of 5 × 10^5^ cells per well. When cells had grown to 70–80% confluence, 500 ng shRNA plasmids were transfected into the cells.

### 2.5. RNA isolation and quantitative real-time reverse transcription PCR

Cells were washed twice with phosphate-buffered saline (PBS), and total RNA was extracted using the TRIzol reagent (Invitrogen, Waltham, MA, United States; 15,596,018) according to the manufacturer’s instructions. cDNA was synthesized from 200 ng of total RNA using a PrimeScript™ RT reagent Kit with gDNA Eraser (Takara, Dalian, China; RR047A), according to the manufacturer’s protocol. The synthesized cDNA was subjected to quantitative real-time PCR (qPCR) using TB Green® Premix Ex Taq™ (Tli RNaseH Plus) (Takara; RR420A). All primers are listed in Table 1. The reaction conditions were as follows: denaturation at 95°C for 30 s; followed by 45 cycles of 95°C for 10 s and 60°C for 30 s; and then melt curve analysis. The sequences were obtained from the National Center for Biotechnology Information (NCBI), and Primer-Blast online software was used to design the related primers. The *ACTB* gene (encoding β-actin) was used as an internal control for each experiment. Dissociation curve analysis was performed after each assay to ensure specific detection. Target genes’ threshold cycle (CT) values and the differences in their CT values (ΔCT) were determined. Relative transcription levels of target genes were presented as fold changes relative to the respective controls using the 2^-ΔΔCT^ threshold method (Livak and Schmittgen, 2001).

**Table 1:** qPCR primer sets for cytokine genes used in this study.

Gene	Forward primer (5′-3′)	Reverse primer (5′-3′)
*PEDV N*	GCAAAGACTGAACCCACTAAT	GCCTCTGTTGTTACTTGGAG
*IFNB*	TAGGCGACACTGTTCGTGTTG	CAAGCAAGTTGTAGCTCATG
*IFNL1*	ATGGCTACAGCTTGGATCGT	TGTGGTGGGCTTGAAAGTGG
*IFNL3*	CCAGTTCAAGTCTCTGTCC	AGTTCCAGTCCTCCAAGA
*OAS1*	GGTTGTCTTCCTCAGTCCTC	GCCTGGACCTCAAACTTC
*ISG15*	GCACAGCAATCATGAGTGAG	GGCCTGTATGTTGCACATCG
*ACTB*	GGACTTCGAGCAGGAGATGG	AGGAAGGAGGGCTGGAAGAG

### 2.6. Western blotting analysis

Cells were lysed at the indicated times in Cell lysis buffer for Western blotting (Beyotime; P0013) supplemented with 1 mM phenylmethanesulfonyl fluoride on ice for 20 min. The cell lysates were then sonicated and centrifuged at 4°C at 12,000 *× g* for 5 min to remove insoluble components. Proteins were resolved using sodium dodecyl sulfate polyacrylamide gel electrophoresis and transferred onto a transfer membrane (Millipore, Billerica, MA, United States; IPVH00010). The membranes were blocked with 5% nonfat dry milk in Tris-buffered saline-Tween for 2 h and then incubated with the primary antibodies at 4°C overnight. The membranes were then incubated with HRP-conjugated secondary antibodies for 1 h at 37°C. The immunoreactive proteins were visualized using an ECL substrate solution (NCM Biotech, Newport, RI, USA; P10200), and the protein bands were quantified using ImageJ software (National Institutes of Health, Bethesda, MD, USA).

### 2.7. Immunofluorescence assay

IPEC-J2 cells were infected with PEDV and treated with butyrate, as described above. The culture supernatant was collected at 24 h and stored at –80°C. The titer of PEDV was measured by limiting dilution on monolayered Vero cells and expressed as the median tissue culture infectious dose (TCID_50_/ml). Infected cells were washed with PBS, fixed with 4% paraformaldehyde fix solution (Beyotime; P0099) for 20 min at room temperature, and then washed three times with PBS. Fixed cells were incubated with PBS containing 0.1% Triton X-100 at room temperature in the dark. Next, the cells were rinsed with PBS and blocked with 1% bovine serum albumin in PBS for 30 min at 37°C and then incubated with anti-N polyclonal antibodies diluted in PBS overnight at 4°C. After three washes with PBS, the Alexa Fluor 488-conjugated goat anti-rabbit IgG (H + L) was used as the secondary antibody, which was incubated with the cells at 37°C for 1 h. After washing with PBS, the fluorescence was examined using fluorescent microscopy (Leica, Wetzlar, Germany) at a magnification of 40 × .

### 2.8. Statistical analyses

Data are represented as the mean ± standard deviation (SD) when indicated, and Student’s *t*-test was used for all statistical analyses, which were completed using GraphPad Prism 5.0 software (GraphPad Inc., La Jolla, CA, United States). Differences between groups were considered significant when the *p*-value was less than 0.05. Unless indicated otherwise, the experiments were performed in triplicate (*n* = 3).

## 3. Results

### 3.1. The inhibitory effect of SCFA on PEDV infection

**Figure 1:**
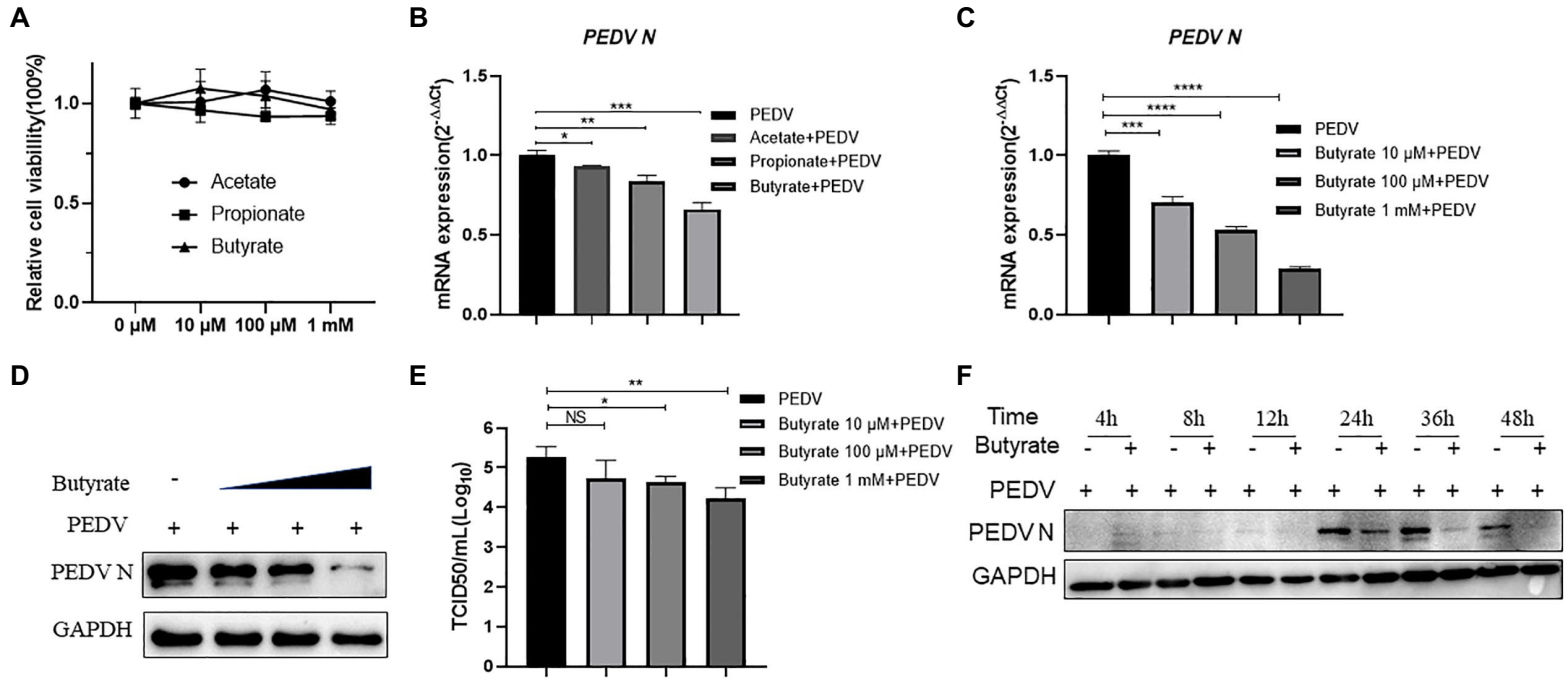
Butyrate pre-treatment protects IPEC-J2 against PEDV infection. **(A)** Effect of acetate, propionate, and butyrate on IPEC-J2 cells. Compounds at concentrations of 0 μM, 10 μM, 100 μM, and 1 mM were added to IPEC-J2 cells and cultured for 48 h. **(B)** IPEC-J2 cells were pretreated with acetate, propionate, and butyrate at 500 μM for 24 h and then infected with PEDV. **(C,D)** Butyrate inhibits PEDV infection. IPEC-J2 cells were pretreated with different concentrations of butyrate (10 μM, 100 μM, and 1 mM) for 24 h and then infected with PEDV (0.1 MOI) for 24 h in the presence of butyrate. **(C)** PEDV viral load detected using qPCR. **(D)** PEDV N protein levels detected using western blotting. **(E)** Viral titers determined using a TCID_50_ assay. **(F)** PEDV N protein levels detected by western blotting at different time points.

SCFAs, mainly acetate, propionate, and butyrate, affect viral infection (Alwin and Karst, 2021). To investigate the effect of SCFAs on IPEC-J2 cells, we initially performed CCK-8 assays to determine the highest noncytotoxic concentration of SCFAs (Figure 1A). Acetate, propionate, and butyrate did not significantly reduce the viability of IPEC-J2 cells at concentrations up to 1 mM. IPEC-J2 cells grown in 12-well plates were not treated or pre-treated with acetate, propionate, and butyrate with 500 μM for 24 h in RPMI Medium 1,640 containing 10% FBS. The culture was kept in an incubator with 5% CO2 at 37°C. Cells were harvested 24 h after infection with PEDV at multiplicity of infection (MOI) of 0.1. The results revealed that butyrate decreased the PEDV RNA level to a greater extent than acetate and propionate (Figure 1B). Further experiments showed that PEDV RNA levels were inhibited by butyrate in a concentration-dependent manner (Figure 1C). Western blotting showed that the level of the PEDV N protein decreased significantly under different concentrations of butyrate (Figure 1D). Similarly, reduction of the viral titer was observed when IPEC-J2 cells were treated with butyrate (Figure 1E). Time point experiments indicated that PEDV N protein levels decreased at 4 hpi, 8 hpi, 12 hpi, 24 hpi, 36 hpi, 48 hpi (Figure 1F). These results revealed the significant antiviral activity of butyrate against PEDV replication.

### 3.2. Butyrate treatment facilitates IFN and downstream ISG expression during PEDV infection

**Figure 2:**
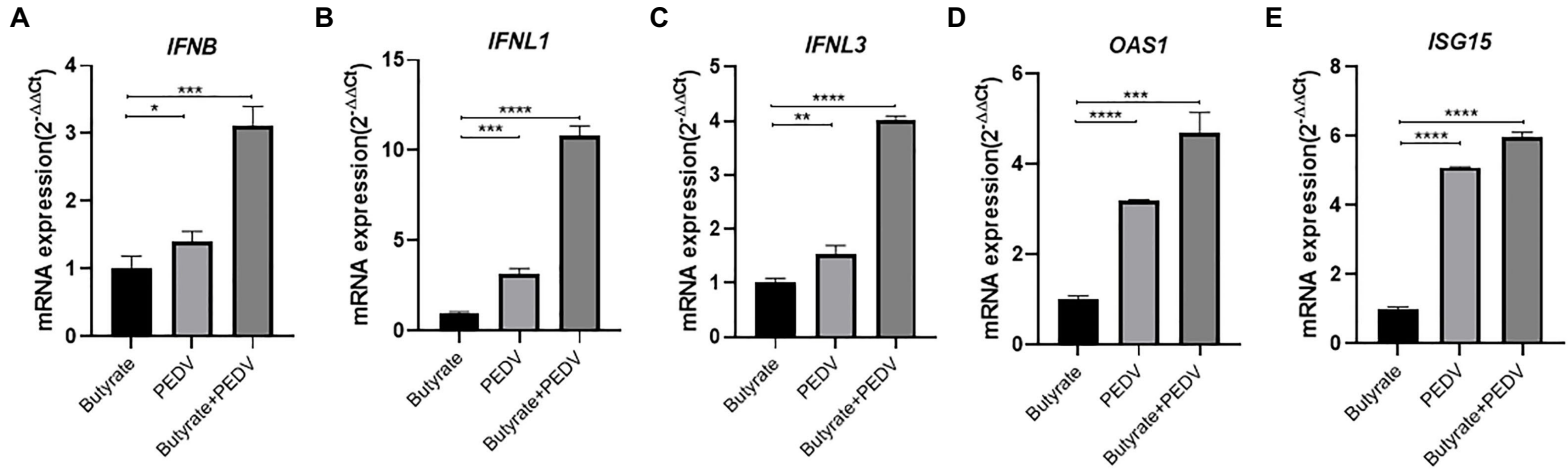
Butyrate treatment mediates an IFN response and ISG expression in PEDV-infected IPEC-J2 cells. **(A)** *IFNB* expression in butyrate-treated and PEDV-infected IPEC-J2 cells after 24 h. **(B)** *IFNL1* expression in butyrate-treated and PEDV-infected IPEC-J2 cells after 24 h. **(C)** *IFNL3* expression in butyrate-treated and PEDV-infected IPEC-J2 cells after 24 h. **(D)** *OAS1*expression in butyrate-treated and PEDV-infected IPEC-J2 cells after 24 h. **(E)** *ISG15* expression in butyrate-treated and PEDV-infected IPEC-J2 cells after 24 h.

Interferon production reduced the replication of PEDV in epithelial cells, decreasing the amount of enterovirus infection and mortality in piglets (Deng et al., 2019). It was also reported that butyrate can activate interferon signaling, ultimately affecting viral infection (Wirusanti et al., 2022). Based on our results showing that butyrate inhibits PEDV, we investigated whether the induction of interferon modulated this phenomenon. We tested the mRNA levels of *IFNB* (encoding IFNβ), *IFNL1* (encoding IFNλ1), and *IFNL3* (encoding IFNλ3) in IPEC-J2 cells infected with the virus. Interestingly, the results showed that butyrate treatment markedly induced the expression of the type I and type III interferons in IPEC-J2 cells (Figures 2A–C). Recently, several studies have indicated that virus infection, especially PEDV infection, in epithelial cells stimulates the secretion of IFN, which triggered high levels of interferon-stimulated genes (ISGs), mainly *OAS1* (encoding 2′-5’-Oligoadenylate Synthetase 1) and *ISG15* (encoding interferon-stimulated gene 15) (Palma-Ocampo et al., 2015; Li et al., 2017, 2021). Consequently, we further verified the expression levels of ISGs downstream of IFN. IPEC-J2 cells were pretreated with 1 mM butyrate for 24 h and infected with PEDV for 24 h. ISG expression at 24 h post-butyrate treatment was assessed using qPCR. As shown in Figures 2D,E, butyrate treatment significantly increased the expression of *OAS1* and *ISG15*.

### 3.3. Butyrate inhibits PEDV replication *via* GPR43 and NF-κB In IPEC-J2 cells

**Figure 3:**
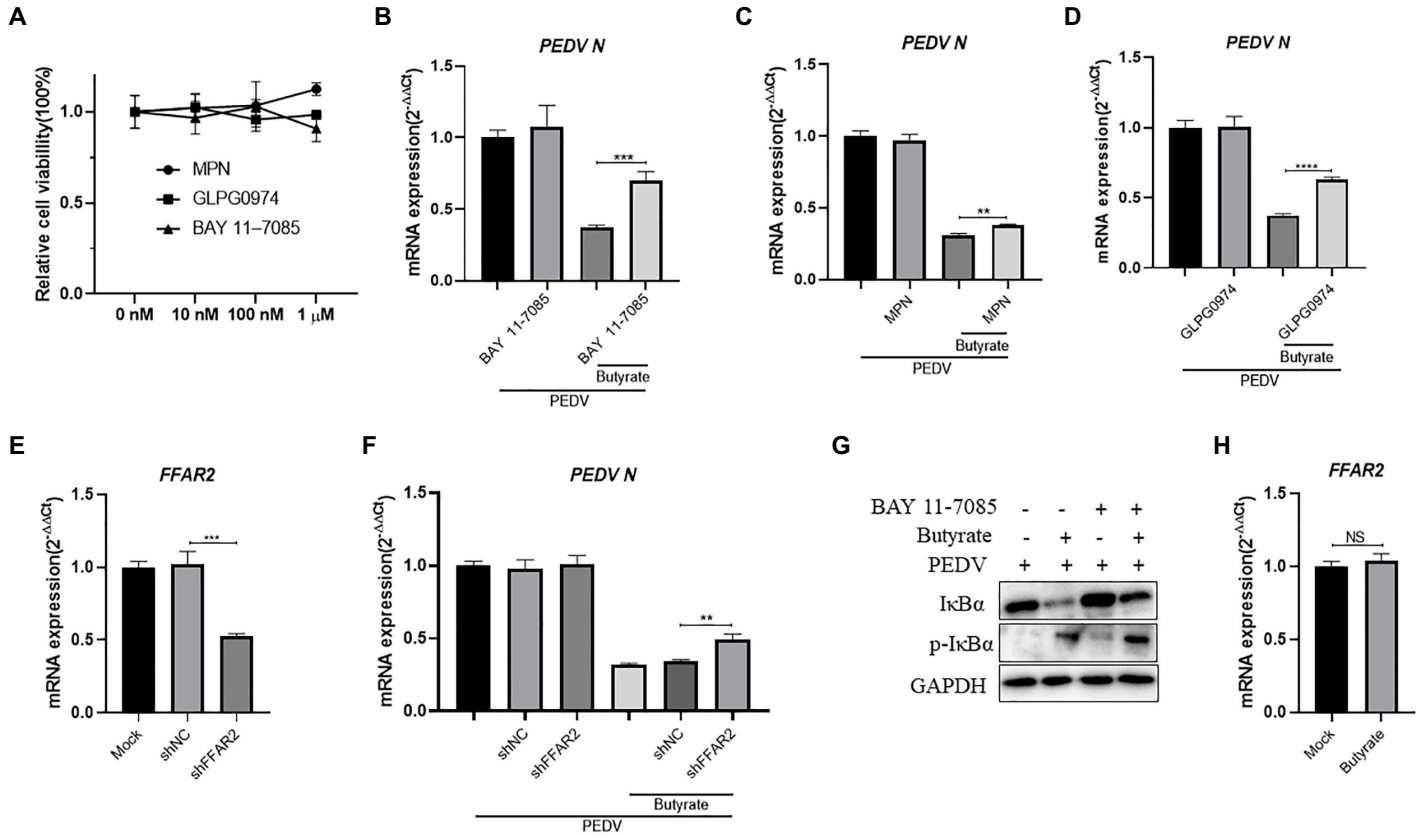
Butyrate inhibits PEDV replication *via* GPR43 and NF-κB in IPEC-J2 cells. **(A)** The effect of MPN, GLPG0974, and BAY 11–7,085 on IPEC-J2 cell. Compounds at concentrations of 0 nM, 10 nM, 100 nM, and 1 μM were added to IPEC-J2 cells and cultured for 48 h. Cell viability was evaluated using the CCK-8 assay. **(B)** IPEC-J2 cells were treated with 1 mM butyrate and 500 nM BAY 11–7,085 for 24 h and then infected with PEDV. PEDV RNA levels were detected using qPCR. **(C)** IPEC-J2 cells were treated with 1 mM butyrate and 1 μM MPN for 24 h and then infected with PEDV. PEDV RNA levels were detected using qPCR **(D)** IPEC-J2 cells were treated with 1 mM butyrate and 500 nM GLPG0974 for 24 h and then infected with PEDV. PEDV RNA levels were detected using qPCR. **(E)** IPEC-J2 cells were transfected with *FFAR2* shRNA plasmids for 24 h. *FFAR2* mRNA expression levels were detected using qPCR. shNC, control shRNA; shFFAR2, shRNA against *FFAR2*. **(F)** IPEC-J2 cells were transfected with *FFAR2* shRNA plasmids and pretreated with butyrate for 24 h. PEDV RNA levels were detected using qPCR. **(G)** IPEC-J2 cells were treated with 1 mM butyrate and 500 nM BAY 11–7,085 for 24 h and then infected with PEDV. Effects of BAY 11–7,082 on virus-induced phosphorylation of IκBα. Protein levels detected by western blotting. **(H)** IPEC-J2 cells were treated with 1 mM butyrate for 24 h. FFAR2 mRNA expression levels were detected using qPCR.

It was reported that SCFAs could activate G-protein coupled receptors and downstream NF-κB against virus infection, such as GPR41, GPR43, and GPR109a (Feng et al., 2018; Trompette et al., 2018; Antunes et al., 2019). The NF-κB signaling pathway has been proven to play a key role during PEDV infection and can lead to interferon activation (Zhang et al., 2018). To investigate the potential mechanism, we investigated the role of NF-κB in butyrate-mediated inhibitory effects on PEDV infection. The cytotoxicity of MPN, GLPG0974, and BAY 11–7,085 in IPEC-J2 cells was first evaluated using CCK-8 assays (Figure 3A). IPEC-J2 cells were pre-treated with the NF-κB pathway inhibitor BAY 11–7,085 and infected with PEDV for 24 h. We found that BAY 11–7,085 abolished the protective effect of butyrate on IPEC-J2 cells against PEDV infection (Figure 3B).

GPR109a was identified as a butyrate receptor (Thangaraju et al., 2009). To further determine whether GPR109a is involved in regulating IFN production, IPEC-J2 cells infected with PEDV were pretreated with MPN, a GPR109a blocker. The results showed that MPN did not significantly recover PEDV infection in butyrate-treated cells (Figure 3C). As an antagonist, GLPG0974 was discovered to inhibit GPR43 effectively (Pizzonero et al., 2014). We found that GLPG0974 recovered PEDV replication in butyrate pretreated IPEC-J2 cells (Figure 3D). We transfected with *FFAR2* (*GPR43*) shRNA plasmids to knockdown *FFAR2* expression in IPEC-J2 cells (Figure 3E) and then pretreated the cells with butyrate. Silencing *FFAR2* abolished the viral inhibition effect of butyrate in PEDV-infected IPEC-J2 cells (Figure 3F), which confirmed the specific activation of GPR43 by butyrate treatment. We investigated whether butyrate mediated NF-κB activation, and we that butyrate treatment activated the phosphorylation of IκBα in IPEC-J2 cells infected with PEDV (Figure 3G). IPEC-J2 cells were only treated with butyrate and has little effect on *FFAR2* expression (Figure 3H). Overall, these results indicated that GPR43, but not GPR109a, was related to the effect of butyrate on PEDV infection, i.e., butyrate activated GPR43 to enhance the expression of IFN in IPEC-J2 cells.

### 3.4. Inhibition of GPR43 and NF-κB reduces the expression of IFN and ISG in butyrate-treated IPEC-J2 cells

**Figure 4:**
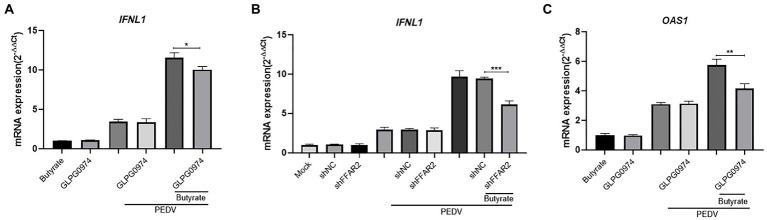
Butyrate inhibits IFN and ISG *via* GPR43 in IPEC-J2 cells. **(A)** IPEC-J2 cells were treated with 1 mM butyrate and 500 nM GLPG0974 for 24 h and then infected with PEDV. *IFNL1* levels in butyrate-treated and PEDV-infected IPEC-J2 cells were assessed after 24 h. **(B)** IPEC-J2 cells were transfected with *FFAR2* shRNA plasmids and pretreated with butyrate for 24 h. *IFNL1* levels in butyrate-treated and PEDV-infected IPEC-J2 cells were assessed after 24 h. **(C)** *OAS1* gene expression in IPEC-J2 cells treated with 1 mM butyrate and 500 nM GLPG0974 and then infected with PEDV for 24 h.

We further examined the expression of IFNs in GLPG0974-pretreated cells during virus infection. The IFN levels were reduced when GLPG0974 pretreated IPEC-J2 were infected with PEDV (Figure 4A). Identically, knockdown of *FFAR2* in IPEC-J2 cells significantly reduced the level of *IFNL1* (Figure 4B). In addition, the induction of *OAS1* in IPEC-J2 cells depended on GPR43 activation (Figure 4C). These results indicated that GPR43 controls *IFNL1* and *OAS1* production, which protects the host against viral infection.

## 4. Discussion

**Figure 5:**
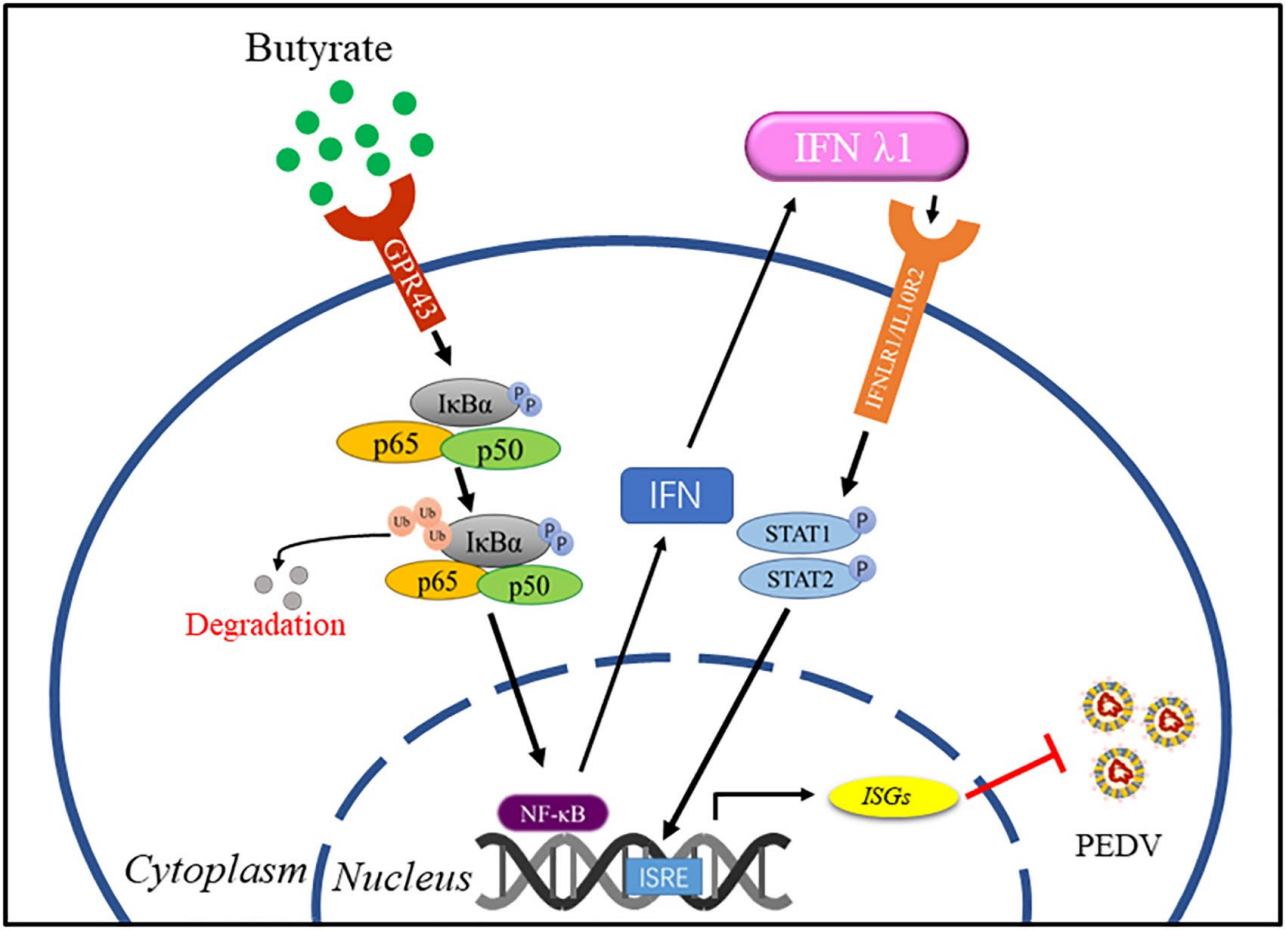
A model for regulation of anti-PEDV innate immunity by butyrate. Butyrate through activation of GPR43 and NF-κB, leading interferon responses and induction of downstream antiviral genes inhibition of PEDV replication.

PED remains a serious health problem and a key economic issue in pig farming. Vaccines for PEDV provide limited protection; therefore, it is necessary to explore new antiviral strategies. SCFAs, as metabolic byproducts, contribute to host physiology and pathology (van der Hee and Wells, 2021). In the present study, we explored the role of the free fatty acid GPR receptor, GPR43, in PEDV infection using its agonist butyrate to evaluate the potential of this receptor as a target for treatment during PEDV infection in intestinal epithelial cells. We identified that butyrate through activation of GPR43 and NF-κB, leading interferon responses and induction of downstream antiviral genes inhibition of PEDV replication (Figure 5).

A study indicated decreased diarrhea incidence in weaned piglets fed with butyrate (Huang et al., 2015). SCFAs positively protect the host from viral infection and reduce the damage to the host after viral infection (Sumbria et al., 2020). For this reason, we investigate the role of an SCFA, butyrate, in the process of PEDV infection. Butyrate at concentrations of 0.01–1 mM promoted IPEC-J2 viability. Chemudupati et al. (2020) demonstrated that butyrate at a high concentration (5 mM) promoted Influenza A virus, reovirus, and HIV-1 infection and replication. By contrast, in the present study, concentrations beyond 1 mM affected cell viability. Our work provides *in vitro* evidence that PEDV replication was inhibited when IPEC-J2 cells were pretreated with sodium butyrate at different concentrations, and PEDV N levels were reduced in a butyrate dose-dependent manner. These data indicated that butyrate negatively affects PEDV.

In the early phase of virus infection, interferon exerts antiviral activities by mediating the innate immune response. At the same time, hundreds of ISGs are activated, which provide antiviral status to the host (Chen and Ling, 2019). Type I and type III interferons induced similar immune responses regarding cell type and signaling kinetics. The type III interferon response shows low efficacy, slow speed, and long-term immunity. *In vitro*, the two types of interferon work in essentially the same way. However, *in vivo*, in response to respiratory and gastrointestinal viral infection, the effects of type III interferon mainly act on epithelial cells (Ingle et al., 2018; Lazear et al., 2019). During PEDV infection of intestinal epithelial cells, IFN λ plays a key role in antiviral activity (Li et al., 2019). It was reported that oral delivery of acetate protected mice from RSV infection by activating IFN-β responses and increasing ISG expression (Antunes et al., 2019). Thus, type I and type III IFNs can effectively protect against viral infections, and several studies revealed that IFNs inhibit PEDV infection (Li et al., 2017; Dong et al., 2022; Xu et al., 2022). The gut microbiota can stimulate type III interferon receptors of intestinal epithelial cells to activate IFN λ1, which can protect the host from viral infection by inducing ISGs (Van Winkle et al., 2022). Comparative transcriptomic and proteomic analyses clearly showed that IFN λ1 is an effective ISG inducer, which upregulated *OAS1* expression, a validated antiviral ISG (Zhao et al., 2020). Our study found that butyrate modulated the type III interferon response to a greater extent than the type I interferon response. In addition, we showed that butyrate treatment induced the transcription of the antiviral ISG, *OAS1*, in intestinal epithelial cells. *OAS1* was shown to decrease PEDV replication, a mechanism that might explain the effects of butyrate (Deng et al., 2019).

The transcription factor NF-κB has an important role in innate immune responses by inducing the expression of interferon and pro-inflammatory cytokines (Xu et al., 2021). PEDV evades host innate immunity to achieve replication *in vivo*, which might be an evolutionary strategy for viral proliferation. Previous studies have demonstrated the following mechanisms of viral disruption of the NF-κB pathway: (a) Nuclear translocation of NF-κB p65 was prevented by inhibiting IκB degradation and phosphorylation (Zhang et al., 2017); and (b) cleavage of NF-κB essential modulator (NEMO) (Wang et al., 2016). Type I interferon and type III interferon require the same transcription factor activation; however, the NF-κB pathway is more important for the production of type III interferon than type I interferon. NF-κB participates in IFN λ1 production (Thomson et al., 2009). In this work, butyrate treatment caused type III interferon production. Based on this result, we investigated whether butyrate mediated NF-κB activation, and the results indicated that pre-treatment with an NF-κB pathway inhibitor decreased IFN λ1 expression in IPEC-J2 cells infected with PEDV.

It has been reported that butyrate can alleviate diarrhea in weaned piglets by regulating the expression of intestinal tight junction protein *via* activating GPR109a (Feng et al., 2018). Zhao et al. (2020) proposed that butyrate alleviates rotavirus-induced epithelial cell apoptosis through GPR109a. However, we examined the expression of GPR109a in cells pretreated with a GPR109a inhibitor, and found that GPR109a had no significant effect on PEDV infection. Moreover, we demonstrated that GPR43 signaling regulated PEDV infection and pretreatment with butyrate resulted in IFN λ1 activation. Butyrate can participate in the barrier function of intestinal epithelial cells. On the one hand, it is beneficial to maintain hypoxia inducing factor (HIF), which is a transcription factor coordinating barrier protection (Kelly and Colgan, 2016). On the other hand, intestinal epithelial cells can produce antimicrobial peptides (AMPs) to regulate intestinal homeostasis, and butyrate can activate GPR43 to promote the production of AMPs (Zhao et al., 2018). We demonstrated that blockade of GPR43 using its antagonist GLPG0974 eliminated the protective effect of butyrate, suggesting that GPR43 mediates the antivirus function of butyrate.

In conclusion, our data suggested that butyrate provides protection from PEDV infection in intestinal epithelial cells. This mechanism involves activation of the innate immune response by GPR43. Our results suggest a strategy involving the inhibitory effect of G protein-coupled receptors on PEDV infection.

## Data availability statement

The original contributions presented in the study are included in the article/supplementary material, further inquiries can be directed to the corresponding authors.

## Author contributions

HH conducted experiments, analyzed data, and wrote final manuscript. XF participated in experiments and formal analysis. HG, HS, and CZ helped with experimental techniques. BZ, NW, and ZL prepared materials. JZ, ML, and LG revised the manuscript and funded this project. All authors contributed to the article and approved the submitted version.

## Funding

This work was supported by the Key-Area R&D Program of Guangdong Province (2020B0202080004), the National Natural Science Foundation of China (31302101), the Science and Technology Planning Project of Guangzhou (202103000096 and 202002030456), the Special Fund for Scientific Innovation Strategy-construction of High-Level Academy of Agriculture Science-Prominent Talents (R2020PY-JC001), the Science and Technology Planning Project of Guangdong Province (2020A1515010950 and 2021A1515011125), and the Start-up Research Project of Maoming Laboratory (2021TDQD002), and the Open Competition Program of Top Ten Critical Priorities of Agricultural Science and Technology Innovation for the 14th Five-Year Plan of Guangdong Province (2022SDZG02).

## Conflict of interest

The authors declare that the research was conducted in the absence of any commercial or financial relationships that could be construed as a potential conflict of interest.

## Publisher’s note

All claims expressed in this article are solely those of the authors and do not necessarily represent those of their affiliated organizations, or those of the publisher, the editors and the reviewers. Any product that may be evaluated in this article, or claim that may be made by its manufacturer, is not guaranteed or endorsed by the publisher.
